# The Effect of Improving Preoperative Sleep Quality on Perioperative Pain by Zolpidem in Patients Undergoing Laparoscopic Colorectal Surgery: A Prospective, Randomized Study

**DOI:** 10.1155/2022/3154780

**Published:** 2022-01-11

**Authors:** Zhennan Xiao, Bo Long, Zeji Zhao

**Affiliations:** ^1^Department of Anesthesiology, Shengjing Hospital of China Medical University, Shenyang, Liaoning, China; ^2^Department of Anesthesiology, Shenzhen Traditional Chinese Medicine Hospital, Shenzhen, Guangdong, China

## Abstract

**Methods:**

A prospective, randomized study was conducted with 88 patients undergoing laparoscopic colorectal surgery. The experimental group (S group, *n* = 44) was given 10 mg of zolpidem tartrate one night before the surgical procedure, while no medication was given to the control group (C group, *n* = 44). The primary outcome was the intraoperative remifentanil consumption. Sufentanil consumption, average patient-controlled analgesia (PCA) effective press times, the visual analog scale (VAS) scores, and incidences of postoperative nausea and vomiting (PONV) were recorded at 6 h (T1), 12 h (T2), and 24 h (T3) postoperatively.

**Results:**

The intraoperative remifentanil consumption was significantly lower in the S group than that in the C group (*p* < 0.01). Sufentanil consumption at 6 h and 12 h postoperatively was significantly lower in the S group than that in the C group (*p* < 0.05); average PCA effective press times and VAS scores, at 6 h and 12 h postoperatively, were significantly lower in the S group than those in the C group (*p* < 0.01); differences between groups 24 h postoperatively were not significant. No significant between-group difference was noted in the incidence of nausea and vomiting.

**Conclusion:**

Improving patients' sleep quality the night before surgical procedure by zolpidem can decrease the usage of intraoperative analgesics and reduce postoperative pain.

## 1. Introduction

Colorectal cancer, with a 5-year survival rate of 32%, is the fifth leading cause of death in the Chinese population [1]. It is among the top 3 cancers that cause the world's total disability-adjusted life year (DALY) disease burden (the other 2 are lung cancer and breast cancer) [2]. In China, the incidence of colorectal cancer increases significantly with age, and older individuals have more difficulty adjusting their sleep to environmental changes [3, 4]. Besides, the presence of sleep disturbance is highly prevalent among the elderly [5]. It is well known that sleep patterns change with age [6] and vary in patients with cancer [7]. Further, cancer and cancer treatments are factors contributing to sleep disturbance [8]. Sleep disturbance is a symptom characterized by impaired sleep quality with abnormal sleep duration [9]. It can lead to poor sleep quality [10], which is considered a risk factor for many conditions, including cardiovascular disease, dementia, obesity, diabetes, depression, pain, and mortality [11–15].

Recent progress has been made in the study of the relationship between preoperative sleep quality and postoperative pain [16–19]. For instance, sleep quality is linked to the development of chronic pain [20, 21]. Sleep deprivation and sleep disruption can lead to pain and hyperalgesia [22–26]. In addition, many studies have shown that pain and sleep have bidirectional interactions; that is, pain may lead to sleep disturbances that result in poor sleep quality, and, in turn, sleep disturbances can cause increased pain and hyperalgesia [22, 27, 28]. Further, some studies found that sleep disturbance may be a stronger predictor of pain than pain is a predictor of sleep [24, 29–32]. Despite ample evidence that postoperative analgesia can improve sleep quality [33–36], there is limited evidence that an improvement in sleep quality contributes to pain relief.

The aim of this prospective, randomized, single-center study was to evaluate the effect of improving preoperative sleep quality on perioperative pain by administering zolpidem. We hypothesized that administration of zolpidem the night before the procedure would improve preoperative sleep quality, reduce intraoperative anesthetic medication, and contribute to postoperative pain relief.

## 2. Materials and Methods

### 2.1. Study Design

The study was approved by the Human Research Ethical Committee of Shengjing Hospital, Shenyang, Liaoning Province, China (IRB registration number: 2018PS385K) and registered at https://www.chictr.org.cn (ChiCTR1900022235, March 31, 2019). It was conducted in accordance with the Declaration of Helsinki. Written informed consent was obtained from all participants. This randomized, single-blind study was conducted at the Department of Anesthesiology at Shengjing Hospital of China Medical University, a tertiary referral hospital.

We included consecutive adult patients (age range, 40–70 years) classified as American Society of Anesthesiologists (ASA) physical status I or II who underwent laparoscopic colorectal surgery between March 2019 and October 2019. Exclusion criteria were patient age <40 years or >70 years; Pittsburgh Sleep Quality Index (PSQI) score ≤7 points; and history of sleep apnea, drug allergy, drug abuse, hepatorenal dysfunction, psychosis, or neuropathy. Patients with a severe hearing impairment or an inability to comprehend or complete the PSQI questionnaire were likewise excluded because of possible barriers to communicating with the anesthesiologists.

### 2.2. Study Intervention

An anesthesiologist, who did not participate in the surgical procedure, used the Pittsburgh Sleep Quality Index (PSQI) to assess each patient's sleep quality. The PSQI is a self-reported questionnaire used in the measurement of sleep quality over a month [37]. The questionnaire contains 19 items that consist of seven components (subjective sleep quality, sleep latency, sleep duration, sleep efficiency, sleep disturbances, use of sleeping medication, and daytime dysfunction). The score ranges from 0 (no difficulty) to 3 (severe difficulty) for each component. Therefore, overall PSQI scores range from 0 to 21, with a higher score indicating a worse sleep quality. A global PSQI score >7 was the cut-off number to indicate poor sleep quality in a Chinese population [38].

All patients with a global PSQI score >7 were enrolled in this study and randomly assigned to either the S (zolpidem tartrate) or C (blank control) group by an investigator, with no clinical involvement, using a head-tail binary result coin toss. The head side of the coin indicated the S group (zolpidem tartrate), and the text or tail side of the coin indicated the C group (blank control). Allocation concealment was ensured by concealing assignments into sealed opaque envelopes that were numbered sequentially and opened the night before the procedure. Zolpidem tartrate (Sanofi Pharmaceutical Co., Ltd., Hangzhou, China) 10 mg was given to patients in the S group the night before the surgical procedure by a nurse independent of the study, while C group patients were not given medication. The patients in each group were blinded to the existence of the other group. Research personnel were maintained blinded throughout the study.

On arrival in the operating room, all patients were given the Leeds Sleep Evaluation Questionnaire (LSEQ) to evaluate their sleep quality the previous night. The LSEQ is a self-rating scale comprising 10 questions, scored on a 100 mm visual analog scale, that pertain to 4 domains: the ease of getting to sleep (GTS, items 1–3), the quality of sleep (QOS, items 4 and 5), waking from sleep (AFS, items 6 and 7), and behavior following wakefulness (BFW, items 8–10) [39]. LSEQ is a suitable tool for comparative assessment of the effect of the drug intervention on sleep quality over a short time. It is a reliable and sensitive measurement of the effects of hypnotics on sleep and early morning behavior [40]. Cronbach's alpha coefficients ranged between 0.78 and 0.92 [41]. The LSEQ has proven reliable and sensitive in numerous studies involving people with insomnia aged 55 and older [42]. Patients were self-rated by placing a mark on the line, with the position of the mark indicating the extent of the change. Higher scores indicate better sleep on a scale of 0 to 100 (corresponding from impairment to improvement). An anesthesiologist who did not participate in the surgical procedure was responsible for recording the LSEQ scores.

### 2.3. Anesthesia Protocol

Intravenous (IV) access and standard monitoring with an electrocardiogram (ECG; lead II), heart rate (HR), blood pressure (BP), and pulse oximetry were established. Peripheral arterial catheters were inserted for intraoperative monitoring of mean arterial pressure (MAP), and a multifunction combination monitor (HXD-1 Beijing Easymonitor Technology Co., Ltd.) was routinely connected to monitor the antinociceptive state and depth of anesthesia.

Once all standard monitors were connected to the patient, general anesthesia was induced by administration of IV sufentanil (0.5 *μ*g/kg) and propofol (1.5 mg/kg). Orotracheal intubation was achieved using a cuffed tube and facilitated by cisatracurium (0.2 mg/kg). Following induction and intubation, ventilation (Drager; Primus, Germany) was performed at tidal volumes of 8 to 10 mL/kg, based on ideal body weight, with a 1 : 2 inspiratory-to-expiratory time ratio. Fresh gas was set to a 50% oxygen-nitrous oxide mixed flow at 2 L/min. Ventilator frequency was initially set at 12 breaths/min and was adjusted as needed to maintain an end-tidal carbon dioxide tension (ETCO_2_), assessed by blood gas analysis, of 35 to 45 mm Hg throughout the procedure.

Remifentanil (1.5–6 *μ*g/kg/h) and propofol (4–10 mg/kg/h), administered continuously by a syringe pump (Zhejiang University Medical Instrument Co., Ltd., Beijing, China), were used for maintenance of general anesthesia. The pain threshold index (PTI) and wavelet index (WLI) were monitored with a multifunction combination monitor (HXD-1 Beijing Easymonitor Technology Co., Ltd.). According to the manufacturer's recommendations, PTI and WLI values of 40 to 60 were maintained to ensure adequate depths of anesthesia during general anesthesia. A single IV bolus of sufentanil (0.1 *μ*g/kg) and ketorolac tromethamine (30 mg IV) was given to all patients 30 min before the end of surgery as prophylaxis against postoperative pain, along with ramosetron hydrochloride (0.3 mg IV) to curb postoperative nausea and vomiting.

After extubation, patients were transferred to the postanesthesia care unit and provided with a patient-controlled analgesia pump (Royal Fornia Medical Equipment Co., Ltd., Shenzhen, China). The pump was prepared at a total volume of 100 ml by mixing 2 *μ*g/kg sufentanil in normal saline. The background infusion rate and a demand dose were 2 ml/h and 1 ml, respectively.

Another anesthesiologist who was blind to the research protocol was responsible for recording the amount of remifentanil consumed during the surgical procedure. A nurse who was blind to the operative details used a visual analog scale (VAS), with 0 corresponding to no pain and 10 to maximum pain, to assess each patient's pain intensity during rest and movement stages at the following postoperative time (*T*) intervals: T1 (6 h), T2 (12 h), and T3 (24 h). The amount of sufentanil consumed within 24 h of the procedure was also recorded at T1, T2, and T3. Average PCA effective press times and incidence of postoperative nausea and vomiting were also recorded within 24 h of the surgical procedure.

### 2.4. Sample Size

According to our previous 20-patient pilot study, the mean intraoperative remifentanil consumption was 0.90 ± 0.35 mg in the patients who received 10 mg of zolpidem tartrate one night before the surgical procedure and 1.20 ± 0.51 mg in the control group. The study was powered to detect a reduction of 0.30 mg in the intraoperative remifentanil consumption. Based on a power of 80% and alpha = 0.05, we calculated that a sample size of 70 (35 in each group) was required. Finally, a sample size of 88 (44 in each group) was required to account for 20% dropout rate.

### 2.5. Statistical Analysis

Descriptive statistics are reported as means with standard deviation (SD) or medians (interquartile range) for continuous variables and as frequencies or proportions for categorical variables. Age, weight, operation time, PSQI score, intraoperative remifentanil consumption, postoperative sufentanil consumption, PCA press times, and VAS scores were compared by the independent *t*-test or Mann–Whitney *U* test; and gender, ASA status, and incidence of postoperative nausea and vomiting were analyzed using either the chi-squared or Fisher's exact test, as appropriate. All statistical analyses were performed with standard software (SPSS v22.0; SPSS Inc., Chicago, IL, USA) and statistical significance was set at *p* < 0.05.

## 3. Results

**Figure 1:**
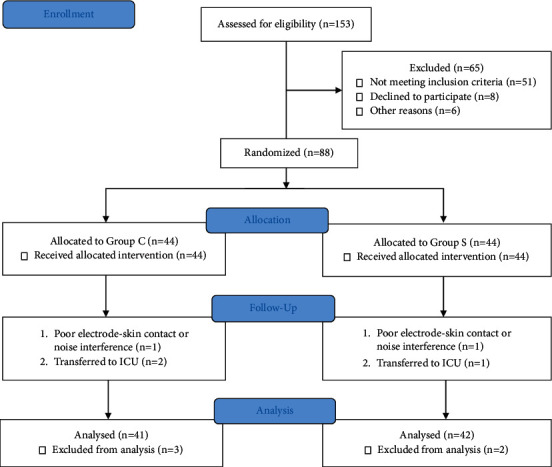
Consolidated standards of reporting trials (CONSORT) flow diagram.

From March 2019 to October 2019, a total of 88 patients were enrolled in the study. The CONSORT diagram for patient recruitment is shown in Figure 1. Two cases were eliminated due to poor electrode-skin contact or noise interference, and another three cases were excluded due to transfer to the ICU. Patient characteristics and intraoperative data were similar in the two groups (Table 1). A significant difference was found in the LSEQ scores on the morning of the surgical procedure. The sleep quality in the S group was improved after administration of zolpidem. Detailed information is shown in Table 2.

Regarding the primary outcome, the intraoperative remifentanil consumption was significantly lower in the S group than in the C group (0.84 [0.61–1.11] vs. 1.00 [0.88–1.55], *p* < 0.01) (Table 3). In the first 24 hours postoperatively, the sufentanil consumption at 6 h and 12 h was significantly lower in the S group than that in the C group (6 h: 17.51 ± 3.09 vs. 20.32 ± 5.07, *p* < 0.05; 12 h: 34.35 ± 5.57 vs. 38.78 ± 8.04, *p* < 0.01), but no between-group difference occurred at 24 h (*p*=0.064) (Table 4). Similarly, average PCA effective press time was significantly lower in the S group than that in the C group at 6 h (1 [1–2] vs. 3 [1–4], *p* = 0.001) and 12 h (2 [1–3.25] vs. 4 [2.5–5], *p* < 0.001) postoperatively, but no between-group difference occurred at 24 h (*p*=0.159) (Table 5). In, addition, patients in the S group had lower VAS scores (*p* < 0.01) at 6 h and 12 h postoperatively compared to the C group, both at rest and during movement (6 h rest: 2.54 ± 0.95 vs. 1.62 ± 0.99, *p* < 0.001; 6 h movement: 3.51 ± 1.36 vs. 2.83 ± 1.15, *p* < 0.01; 12 h rest: 3.78 ± 1.04 vs. 3.00 ± 1.21, *p* < 0.01; 12 h movement: 4.80 ± 1.15 vs. 3.98 ± 1.24, *p* < 0.01). However, the between-group differences at 24 h postoperatively were not significant (24 h rest: *p*=0.066; 24 h movement: *p*=0.069) (Table 6). No significant between-group differences were noted in the incidence of nausea and vomiting between the 2 groups (6 h: *p*=0.625, 12 h: *p*=1, 24 h: *p*=0.738) (Table 7).

## 4. Discussion

The results of this study showed that administering a dose of zolpidem (10 mg) can improve sleep quality preoperatively and reduce the amount of analgesics used intraoperatively and postoperatively. Moreover, lower pain intensity, both at rest and during movement, was noticed in patients given zolpidem compared to patients in the control group within the first 12 h after surgery. However, no significant difference was found in the incidence of PONV and length of hospital stay between treatment and control group patients.

Sleep is a physiological phenomenon which is indispensable to an individual's physical and mental health [43, 44]. However, almost half of older adults in China experience various degrees of sleep disturbance [45, 46]. Previous investigators have speculated that poor sleep quality predicts high next-day pain intensity that may persist up to 2 weeks after surgery [47]. Another published review concluded that behavioral sleep interventions can produce improvements in sleep that are beneficial to pain outcomes in middle-aged or older adults [48]. Consequently, it may be worthwhile to relieve postoperative pain by improving sleep quality.

In the current study, the patients in the S group achieved higher LSEQ scores the following morning after receiving a dose of zolpidem (10 mg). This finding suggests that zolpidem can significantly increase sleep quality in patients with sleep disturbance. Zolpidem, an alternative to benzodiazepines, is a prescription sedative-hypnotic drug with a well-established safety profile [49]. It improves sleep quality by helping patients fall asleep quickly and producing a more natural sleep pattern [50]. Although some studies have reported that hypnotics improve GTS and QOS but produce a dose-dependent “hangover” effect that results in impairment in AFS [37], we did not find any difference in AFS in the S group compared with the C group. A possible explanation is that we only administered a single 10 mg dose of zolpidem one day before the surgical procedure, which is a much lower dosage than in previous studies. Furthermore, zolpidem is one of the nonbenzodiazepine hypnotics with a short half-life and low incidence of residual effects on alertness the following morning.

Regarding the primary outcome, our study revealed that patients with better sleep quality were more likely to have lower analgesics consumption during the surgical procedure. In clinical practice, the administration of antinociceptive analgesics is usually determined by the anesthesiologist's clinical experience [51]. Therefore, it is challenging to reach an appropriate analgesic dosage without effective or objective evaluation tools. To support the proper use of analgesics, we applied PTI to reflect the antinociceptive state in order to guide the administration of opioids under general anesthesia. PTI is a newly developed EEG-derived parameter that can be used to predict the response to, rather than detecting the result of, noxious stimulation. Wu et al. demonstrated that the PTI is proven to be effective and subtle in predicting the nociceptive response induced by noxious stimuli during general anesthesia [52]. The principle of the PTI calculation was reported in China Medical Engineering in 2017 [53]. In this study, lower remifentanil consumption was reported in the S compared to C group. To our knowledge, only limited research has been conducted to show that improved sleep quality can reduce the amount of analgesics administered intraoperatively.

Furthermore, patients with better sleep quality had significantly lower sufentanil consumption, less average PCA effective press times, and lower VAS scores at 6 h and 12 h postoperatively, compared to the C group. Our result is partially consistent with that of a systematic review showing that perioperative administration of sleep-promoting drugs can improve pain relief [54]. Similarly, Shakya et al. found that zolpidem 10 mg, administered from 2 days preoperatively to 5 days after surgery, can improve sleep quality, relieve pain, and promote recovery in patients underwent total hip arthroplasty [55]. Another similar result was reported by Gong et al. in a study involving 148 patients who underwent total knee arthroplasty. They found a greater improvement in sleep quality after taking zolpidem for 2 weeks, with decreased postoperative VAS pain scores and analgesics consumption and a better quality of life [56].

However, there were several differences between their studies and ours. Firstly, in contrast with their studies in orthopedic patients, we mainly focused on the patients with colorectal cancer. Reportedly, nearly 40% of patients with colorectal cancer in China have preoperative insomnia [57]. A case-control study demonstrated that better sleep quality is protective for patients with colorectal cancer [58]. Secondly, patients undergoing laparoscopic colorectal surgery are more subjected to visceral pain rather than somatic pain. Visceral pain is defined as a deep, dull, and vague sensation that often cannot be described or is poorly localized [59]. Acute visceral pain may develop into chronic visceral pain that results in behavioral symptoms such as anxiety, fear, and depression [60]. In addition, opioids are commonly used in the management of acute visceral pain; however, large doses of opioids may induce constipation that is associated with a series of physical and psychological symptoms as well as a poor quality of life [61]. Therefore, it is crucial for patients with colorectal cancer to have better pain relief and a reduction in opioid requirements.

Notably, our finding suggested that improved sleep quality can reduce the amount of analgesics used both intraoperatively and within the first 12 h postoperatively. A possible explanation lies in the mechanisms underlying the relationship between sleep and pain, including endogenous pain modulation, inflammation, affection, and mood as well as some roles of different endogenous substances [62]. Endogenous pain modulation refers to the series of actions that affect nociceptive signal processing central nervous system. Ample clinical evidence shows that sleep fragmentation and deprivation can interfere or even inhibit the descending pain inhibitory system, thus leading to an increase in pain intensity or pain hypersensitivity [63–65]. Moreover, sleep disturbance is associated with central sensitization. Smith et al. demonstrated that sleep disruption enhanced secondary hyperalgesia in males and increased temporal summation in females [66].

Cytokines play critical roles in the development of inflammatory pain. Sleep disturbance can lead to increased levels of proinflammatory cytokines such as IL-1*β*, IL-6, TNF-*α*, and CRP-6, which further increase pain intensity and sensitivity and increase the incidence of pain [67–70]. Additionally, individuals who experience poor sleep report higher negative effect (e.g., anxiety, anger, and fear), which is believed to cause hypervigilance and sensitization to pain [71, 72]. Furthermore, the potential significance of endogenous substances has been elucidated in the mechanism of pain. Roman et al. found that sleep loss can decrease the sensitivity of the serotonin-1A receptor, which is involved in the processing of nociception [73]. Many clinical and experimental studies illustrate that sleep deprivation is related to increased NMDA receptor activation, which is involved in secondary hyperalgesia and temporal summation [74–78]. Sleep deprivation also can downregulate dopamine 2 receptors that are associated with the modulation of acute antinociception [79, 80]. Last, a weak zolpidem-induced analgesic effect through opioidergic mechanisms was observed in a mouse model [81].

Generally speaking, the intraoperative use of remifentanil has been closely linked to postoperative hyperalgesia. Thus, the lower intraoperative remifentanil consumption may contribute to the less postoperative sufentanil consumption and lower pain intensity in the S group, compared to those in the C group. Nevertheless, a study published in the journal, *Anaesthesia* [82], confirmed that it is less likely to cause postoperative hyperalgesia when remifentanil infusion rate is below 0.2 *μ*g·kg^−1^·min^−1^. In our clinical trial, remifentanil infusion rate was 0.08 ± 0.02 *μ*g·kg^−1^·min^−1^ in the C group and 0.07 ± 0.02 *μ*g·kg^−1^·min^−1^ in the S group. Both rates were far less than 0.2 *μ*g·kg^−1^·min^−1^. This led us to believe that the difference in postoperative analgesia was mainly due to the impact of improved sleep quality by zolpidem.

Based on the above research, we infer that zolpidem can improve patients' sleep quality preoperatively. This improvement contributes to the reduction in the usage of opioid analgesics intraoperatively and postoperatively (within 12 h). Further studies with objective measures (e.g., fMRI and PSG) should be performed to validate our findings.

Our study has some limitations. First, sleep quality was only assessed using the PSQI and LSEQ without polysomnography for evaluating sleep quality, quantity, and architecture. We did not assess patient fatigue and depression, which may also influence the measured outcomes. Second, light and noise may influence sleep quality in hospitalized patients. To minimize the effect of such disruptions, we allocated the patients randomly, thereby distributing the potential confounders equally between the groups. Additionally, as this is only a single-center single-blind trial in a relatively small population, a multicenter trial is necessary to verify its results. We acknowledge this was a single-blind study since patients were not adequately blinded. However, patients in each group were blinded to the existence of the other group. In addition, all researchers taking measurements remained blinded to the details of the study, as were also the data analysts. Blinding was also maintained by randomization and allocation concealment to minimize bias. Finally, we only administered zolpidem the night before the surgical procedure and reported the results within the first 24 h postoperatively. We did not examine the long-term duration and efficacy of pain control. Future studies should focus on the suitable dosage of zolpidem and any other medical interventions.

## 5. Conclusion

Improving the sleep quality of patients the night before surgery by zolpidem can decrease the usage of intraoperative analgesics and reduce postoperative pain. Given the widely varying available methods, further exploration of suitable and effective ways to improve sleep quality while minimizing side effects is required. The results of this study may provide a new approach to promoting enhanced recovery after surgery.

## Figures and Tables

**Table 1:** Patient characteristics and intraoperative data.

	Group C	Group S
Gender (M/F)	21/20	23/19
ASA status (I/II)	17/24	16/26
Age (years)	60.34 ± 7.02	60.02 ± 7.32
Weight (kg)	66.83 ± 11.07	65.48 ± 10.71
PSQI	9.22 ± 1.81	8.98 ± 1.62
Operation time (min)	205.29 ± 35.46	192.43 ± 33.36
Hospitalization time (days)	19.90 ± 3.07	20.81 ± 2.04

Note: data are expressed as mean ± SD. Abbreviations: PSQI: Pittsburgh Sleep Quality Index; group C: blank control; group S: zolpidem tartrate.

**Table 2:** Leeds Sleep Evaluation Questionnaire (LSEQ) scores.

	Group C	Group S	*p* value
GTS	45.20 ± 8.71	76.55 ± 7.63^*∗*^	<0.001
QOS	46.44 ± 9.35	74.29 ± 7.46^*∗*^	<0.001
AFS	45.90 ± 13.08	51.48 ± 15.90	0.085
BFW	52.10 ± 12.49	61.76 ± 10.99^*∗*^	<0.001

Note: data are expressed as mean ± SD.  ^*∗*^*p* < 0.05 vs. group C. Abbreviations: GTS: getting to sleep; QOS: quality of sleep; AFS: awakening from sleep; BFW: behavior following wakefulness. Group C: blank control; group S: zolpidem tartrate.

**Table 3:** Intraoperative remifentanil consumption.

	Group C	Group S	*p* value
Remifentanil dose (mg)	1.00 (0.88–1.55)	0.84 (0.61–1.11)^*∗*^	0.002

Note: data expressed as median (interquartile range).  ^*∗*^*p* < 0.05 vs. group C. Abbreviations: group C: blank control; group S: zolpidem tartrate.

**Table 4:** Postoperative sufentanil consumption.

	Group C	Group S	*p* value
Postoperative 6 h (ug)	20.32 ± 5.07	17.51 ± 3.09^*∗*^	0.016
Postoperative 12 h (ug)	38.78 ± 8.04	34.35 ± 5.57^*∗*^	0.004
Postoperative 24 h (ug)	73.68 ± 14.69	68.41 ± 10.58	0.064

Note: data expressed as means ± SD.  ^*∗*^*p* < 0.05 vs. group C. Abbreviations: group C: blank control; group S: zolpidem tartrate.

**Table 5:** Average PCA effective press times.

	Group C	Group S	*p* value
Postoperative 6 h	3 (1–4)	1 (1–2)^*∗*^	0.001
Postoperative 12 h	4 (2.5–5)	2 (1–3.25)^*∗*^	<0.001
Postoperative 24 h	5.68 ± 4.25	4.52 ± 3.10	0.159

Note: data expressed as means ± SD and median (interquartile range).  ^*∗*^*p* < 0.05 vs. group C. Abbreviations: group C: blank control; group S: zolpidem tartrate.

**Table 6:** Postoperative VAS.

	Group C	Group S	*p* value
PO6 (rest)	2.54 ± 0.95	1.62 ± 0.99^*∗*^	<0.001
PO6 (movement)	3.51 ± 1.36	2.83 ± 1.15^*∗*^	0.016
PO12 (rest)	3.78 ± 1.04	3.00 ± 1.21^*∗*^	0.002
PO12 (movement)	4.80 ± 1.15	3.98 ± 1.24^*∗*^	0.002
PO24 (rest)	4 (3.5–5)	4 (3–4)	0.066
PO24 (movement)	5 (4–6)	4 (4–5)	0.069

Note: data expressed as means ± SD and median (interquartile range).  ^*∗*^*p* < 0.05 vs. group C. Abbreviations: PO6: postoperative 6 h; PO12: postoperative 12 h; PO24: postoperative 24 h. Group C: blank control; group S: zolpidem tartrate.

**Table 7:** Nausea and vomiting.

	Group C	Group S
Postoperative 6 h	12	10
Postoperative 12 h	8	8
Postoperative 24 h	5	4

Note: values are *n* (%). Abbreviations: group C: blank control; group S: zolpidem tartrate.

## Data Availability

The data and materials that support the findings of this study are available from the corresponding author upon reasonable request.
